# Hierarchical Structure of Generalized Thermodynamic and Informational Entropy

**DOI:** 10.3390/e20080553

**Published:** 2018-07-25

**Authors:** Pierfrancesco Palazzo

**Affiliations:** Department of Astronautical, Electrical and Energy Engineering (DIAEE), Sapienza Università di Roma, 00184 Roma, Italy; pierfrancesco.palazzo@uniroma1.it; Tel.: +39-333-299-4321

**Keywords:** hierarchical configuration, hierarchical structure, equilibrium, nonequilibrium, first law, second law, generalized thermodynamic entropy, generalized informational entropy, generalized exergy, equipartition theorem of energy, non-equipartition theorem of entropy

## Abstract

The present research aimed at discussing the thermodynamic and informational aspects of entropy concept to propose a unitary perspective of its definitions as an inherent property of any system in any state. The dualism and the relation between physical nature of information and the informational content of physical states of matter and phenomena play a fundamental role in the description of multi-scale systems characterized by hierarchical configurations. A method is proposed to generalize thermodynamic and informational entropy property and characterize the hierarchical structure of its canonical definition at macroscopic and microscopic levels of a system described in the domain of classical and quantum physics. The conceptual schema is based on dualisms and symmetries inherent to the geometric and kinematic configurations and interactions occurring in many-particle and few-particle thermodynamic systems. The hierarchical configuration of particles and sub-particles, representing the constitutive elements of physical systems, breaks down into levels characterized by particle masses subdivision, implying positions and velocities degrees of freedom multiplication. This hierarchy accommodates the allocation of phenomena and processes from higher to lower levels in the respect of the equipartition theorem of energy. However, the opposite and reversible process, from lower to higher level, is impossible by virtue of the *Second Law*, expressed as impossibility of Perpetual Motion Machine of the Second Kind (PMM2) remaining valid at all hierarchical levels, and the non-existence of Maxwell’s demon. Based on the generalized definition of entropy property, the hierarchical structure of entropy contribution and production balance, determined by degrees of freedom and constraints of systems configuration, is established. Moreover, as a consequence of the *Second Law*, the non-equipartition theorem of entropy is enunciated, which would be complementary to the equipartition theorem of energy derived from the *First Law*.

## 1. Premise

The title of the present article addresses both the thermodynamic and informational aspects of entropy concept to propose a unitary perspective of its definitions as an inherent property of any system in any state. However, the treatise is here focused on physical aspects as a prerequisite to extend the conceptual framework to information science to pursue the attempt of achieving an overarching and unitary theory.

On the one side, the term “generalized thermodynamic entropy” addresses the physical aspect of phenomena occurring in any system. On the other side, the definition of “generalized informational entropy” is the corresponding property of interest developed in the domain of Information Science and Geometry. The novelties here proposed concern: (i) the generalization of thermodynamic entropy and its hierarchical structure associated to multi-scale system configuration; and (ii) the possibility (and, for a rigorous approach, the need) of extending to information science the generalization of foundations and properties in the thermodynamic domain with the aim of achieving a complete and consistent conceptual framework. The intent is here to highlight correlations among different facets of the theoretical and methodological building under elaboration by the community of physicists and information scientists.

## 2. Introduction

The following main points represent the context in which the present study is placed:

(i) Thermodynamic foundations framework in the conception of Hatsopoulos, Gyftopoulos and Beretta [1,2,3,4] claim that thermodynamic entropy is an inherent property of matter in its broader sense related to any system, large or small, in any state, equilibrium or non-equilibrium, even at macroscopic non-statistical level with no need for any microscopic statistical rationale [5,6].
(ii) The inherent character of entropy extends its validity to any scale of physical dimensions, hence classical and quantum mechanics equations of any particle are in compliance with the inherent essence and physical meaning of entropy including non-statistical and statistical methods of mechanics and thermodynamics [7]. In addition, quantum thermodynamics and the unified quantum theory of mechanics and thermodynamics [8,9,10,11] have demonstrated that irreducible uncertainties and probabilistic nature of phenomena are the ultimate root causes of irreversibility existing in microscopic dynamics.
(iii) Nevertheless, according to an information-based conception, a different school of thought has devised proofs that information and Shannon informational entropy [12,13] are in turn inherently associated to physic states of matter, as demonstrated by Jaynes [14,15], Landauer [16,17], and Karnani, Paakkonen and Annila [18]. Therefore, Boltzmann and Gibbs statistical entropy are correlated to Shannon entropy and this relationship is not only a formal correspondence and homology. Both thermodynamic aspect and informational aspects are inherent to any system in any state and the implication of quantum mechanics in quantum information theory advocates this principle [19]. Indeed, informational entropy is in turn an inherent property of matter as any physical state is characterized by an amount of information and a corresponding amount of uncertainty that depends on the scale of the system up to quantum where Heisenberg indetermination principle constitutes a physical fundamental. The statement that information is a physical entity does not disprove that entropy is an inherent property of matter. Instead, both represent different expressions of a unique fundamental and elementary characteristic of the phenomenological physical reality.
(iv) Information is an inherent property of any system in any state since it is associated to the state of properties. Consequently, the relationship between thermodynamic and informational viewpoints represent an intrinsic property of any system in any state being the two viewpoints coexisting and complementary. Any microscopic up to macroscopic scale of classical (and non-statistical) thermodynamics is affected by this correlation and generalization of theorems or properties can be adopted in the domain of information theory, as explained by Kafri [20].

The main objective is here to discuss the hierarchy characterizing any system and the subsequent structure of thermodynamic and informational entropy deriving from this multi-level description. The interest relies in extending these findings to achieve a correspondence and equivalence between thermodynamic entropy and informational entropy and their respective role in the description of complex abiotic and or biotic systems.

## 3. Considerations on Physical Aspect of Second Law and Thermodynamic Entropy

One of the paradigms of Thermodynamics conceptual architecture is founded on axiomatic definitions and demonstrations of principles and theorems developed by Keenan, Hatsopoulos, Gyftopoulos and Beretta. The First Law and Second Law have been reformulated and, in their perspective, the Second Law statement asserts the existence and uniqueness of stable equilibrium state for a given system A composed by r constituents, described by s parameters and characterized by a constant energy content [1]. A corollary of this statement is the impossibility of “Perpetual Motion Machine of the Second Kind (PMM2)” which has been adopted to demonstrate an alternative formulation of thermodynamic entropy as a non-conservative, additive and state property. In particular, this definition of entropy property for macroscopic states and processes, in the framework of Classical Thermodynamics, has been founded on its nature inherent to all systems, large or small, in all states, equilibrium and non-equilibrium. On this basis, its definition has been derived replacing the heat interaction Q, appearing in Clausius definition, with the difference between energy E and available energy $\Omega^{R}$ of a system A interacting with an external reference system, or reservoir, R, times a parameter $C_{R}$ characteristic of the reservoir:

(1) $$S_{1}-S_{0}=\frac{1}{C_{R}}\left[\left(E_{1}-E_{0}\right)-\left(\Omega_{1}^{R}-\Omega_{0}^{R}\right)\right]$$

where it is proved that $C_{R}=T_{R}$ and $T_{R}$ is the constant temperature of R [1]. The physical meaning of this expression is that the entropy variation is determined by the amount of non-useful heat released by the system, along whatever process between initial and final states, to the reservoir. Indeed, the energy minus the available energy results into the non-available energy. The available energy is defined with respect to a reference system and hence corresponds to the exergy $EX^{R}$, in turn depending on a fixed reference thermodynamic state, both being additive state properties. Therefore, the above expression of entropy can be turned in the one considering exergy in lieu of available energy $S_{1}-S_{0}=\frac{1}{C_{R}}\left[\left(E_{1}-E_{0}\right)-\left(EX_{1}^{R}-EX_{0}^{R}\right)\right]$. Considering that exergy is defined as the maximum net useful work withdrawable from a system interacting with a reservoir, here the physical meaning is that entropy corresponds to the minimum net non-useful heat released by the system to the reservoir. The consequence, as anticipated, is that the concept of heat interaction Q is not more used in this definition [21,22,23].

To complete the discussion of the physical meaning, this definition assumes that entropy expressed in Equation (1) is an inherent property of matter, hence it does not depend on whatever external reference system or reservoir is assumed [1,2,3,4]. The role of the reservoir is therefore auxiliary only and recent studies demonstrate that entropy can be defined with no use of the reservoir concept [23]. The available energy is replaced by the exergy property, in turn conceived and defined as a non-conservative and additive state property [24,25,26,27] to provide components of exergy associated to all contributions of available energy of a system interacting with a reservoir. The entropy-exergy relationship ensures that the exergy method, adopted in the design and optimization of processes and plants [28], properly accounts for non-equilibrium and irreversible phenomena focused by Second Law analyses.

A summary of the logical rationale underpinning the framework of foundations under discussion is described in the following sequence to highlight the reasons of a more general paradigm:

(1) The Second Law statement is based on the existence and uniqueness of stable equilibrium.
(2) Stable equilibrium implies thermal equilibrium, chemical equilibrium and mechanical equilibrium.
(3) Corollary of stable equilibrium is the impossibility of Perpetual Motion Machine of the Second Kind (PMM2).
(4) PMM2 is adopted in the proof of the entropy definition related to temperature, hence it is the definition of a thermal entropy property.
(5) Highest-(thermal)-entropy principle is applied to prove that stable equilibrium implies the equality of temperature, potential and pressure while thermal entropy determines thermal energy and heat interaction only, this representing a logical incompleteness and inconsistency thus introducing an incongruity [29,30,31].
(6) To remove the incongruity, equality of temperature, potential and pressure have to imply thermal, chemical and mechanical equilibria and this opposite proof needs chemical entropy and mechanical entropy, in addition to thermal entropy, to assert a highest-generalized-entropy principle to be used in the proof [29,30,31].

As entropy requires the concept of exergy, in turn derived from the available energy of the composite system-reservoir, then the formulation of chemical exergy and mechanical exergy, in addition to thermal exergy, is needed to achieve the chemical entropy and mechanical entropy, in addition to thermal entropy, as components of the generalized thermodynamic entropy property, which enables demonstrating the necessity and sufficiency of stable equilibrium for equality of temperature, potential and pressure, thus proving the Second Law with a complete and consistent logical rationale. This paradigm should also be valid at microscopic level, more rigorously at any dimensional scale of matter up to elementary particles obeying to quantum mechanics. For this very reason, the following sections focus on the extension of all components of thermodynamic entropy to each and every mesoscopic level constituting a multi-scale system in the perspective of statistical and quantum physics.

## 4. Second Law Statements Related to Thermal or Chemical Potentials

Among all statements of Second Law reported in the literature, the existence of uniqueness of stable equilibrium of a system constitutes the principle from which the non-existence of an ideal Perpetual Motion Machine of the Second Kind (PMM2) is inferred [1]. PMM2 implies that a system does not exist which is capable of converting a given amount of thermal energy at high temperature into mechanical energy with no production of thermal energy at lower temperature. This represents a statement of the Second Law enunciated by Kelvin and Planck in the sense that the PMM2 undergoes a direct heat-to-work ideal conversion cycle process. Besides, the statement of Clausius addresses to the impossibility of converting thermal energy at low temperature into thermal energy at high temperature with no contribution of mechanical energy input. In this case, the PMM2 can be regarded as undergoing an inverse work-to-heat ideal conversion cycle process. The rationale behind the impossibility of PMM2 is the non-existence of Maxwell’s demon [32]. The concept of Maxwell’s demon, implied with the Second Law, consists of a being or a device capable of selecting and separating particles of any system with higher kinetic or potential energy from particles with lower kinetic or potential energy, with no net effects on the surrounding environment interacting with the system. In other terms, there is no means to select and separate particles at higher velocity from particles at lower velocity and particles at lower relative distance from particles at higher relative distance. If it existed, this selective segregation would be able to revert an irreversible process or to generate the availability of the system equivalent to a reduction of thermodynamic entropy property between the initial non-equilibrium state and the final stable equilibrium state.

The non-existence of PMM2 is adopted to demonstrate the formulation of entropy property as used in the proof developed by Gyftopoulos and Beretta [1], however, the concept of PMM2 can be generalized to chemical potential that depends on particles relative position determining the system geometry, in addition to the thermal aspect that depends on particles relative velocity determining the system kinematics. Hence, considering that mass interaction, occurring in open systems, assumes the same role of heat interaction in closed systems, the Second Law can be regarded in terms of mass interactions in addition to heat interactions. A consequence of the extended types of interactions governed by the Second Law, in the specific case of cyclic processes, is that the definition of entropy property can be expressed in terms of thermal or chemical cycle efficiency. For thermal processes at constant chemical potential and variable pressure, the following canonical expression of heat cycle efficiency applies: $W=Q\cdot \left(1-\frac{T_{R}}{T}\right)=Q\eta^{T}$. On the other side, accounting for chemical processes at constant temperature and variable pressure, the following expression of mass cycle efficiency corresponds to, and is homologous to, the previous one and is based on inter-particle potential energy (chemical potential) in lieu of inter-particle kinetic energy (temperature): $W=M\cdot \left(1-\frac{\mu_{R}}{\mu }\right)=M\eta^{C}$. It is noteworthy that the Phase Rule F=C+2−P ensures at least two independent intensive properties that, in the case of isopotential or isothermal processes, consist of temperature and pressure or potential and pressure, respectively.

The formulation of mass cycle efficiency can be proved adopting the rationale proposed by Gyftopoulos for the heat cycle efficiency [2]. Assuming a reversible process to convert mass interaction into work interaction the balance of energy and entropy relating to the conversion cycle of the internal system is evaluated. The system undergoes an input of mass interaction M at high potential μ associated to chemical entropy input $S^{C}=\frac{M}{\mu }$ to be converted into work interaction W. The entropy balance of the cyclic process requires that an equal amount of chemical entropy output, corresponding to $\frac{M}{\mu }$, be associated to mass interaction released at low potential $\mu_{R}$. However, the release of entropy at $\mu_{R}$ must necessarily be associated to a mass interaction $\mu_{R}S^{C}=\mu_{R}\frac{M}{\mu }$. Hence, the overall interactions balance is $W=M-\mu_{R}\frac{M}{\mu }=M\cdot \left(1-\frac{\mu_{R}}{\mu }\right)$ in which the cycle efficiency corresponds to the formulation assumed: $W=M\cdot \left(1-\frac{\mu_{R}}{\mu }\right)$. The non-completeness of energy transfer occurs after reaction since before reaction no contribution and no Maxwell demon can act to increase the amount of energy transfer [2,3]. Both thermal and chemical aspects of Second Law, according to the above highlighted dualism and symmetry relating to closed and open systems, underpin all definitions of the Second Law in terms of non-existence of PMM2. The set of statements of Second Law accounting for thermal, chemical and mechanical interactions is discussed in the following section.

## 5. Perpetual Motion Machines of Second Kind (PMM2) as a Corollary of Second Law

The Perpetual Motion Machine of the Second Kind (PMM2) is a corollary directly derived from the Second Law enunciated in terms of existence and uniqueness of stable equilibrium. With reference to the previous section, temperature and potential drive those processes occurring in thermal-mechanical PMM2 or in chemical-mechanical PMM2, then the following set of statements can be characterized by the properties involved, categorized by the process occurring and classified in terms of specific PMM2 definition. It is intended that ideal direct or inverse cycle conversions are reversible processes moving the systems through stable equilibrium states.

### 5.1. Thermal-Mechanical PMM2

Thermal–Mechanical aspect relating to heat-to-work or work-to-heat interactions conversion occurring in closed systems can be characterized and categorized as follows:

1. Mechanical aspect of non-existence of PMM2 performing an ideal direct heat-to-work conversion cycle implies that it is not possible to convert a given amount of thermal energy at high temperature into mechanical energy with no production of thermal energy at lower temperature (Kelvin–Planck and Poincaré); in this case, the PMM2 canonical efficiency is $\eta^{\begin{array}{l} DIRECT\\ THERMAL \end{array}}=1-\frac{T_{R}}{T}$.
2. Thermal aspect of non-existence of PMM2 performing an ideal inverse work-to-heat conversion cycle implies that it is not possible to convert a given amount of thermal energy at low temperature into thermal energy at high temperature with no contribution of mechanical energy input (Clausiu and Thompson).
3. Mechanical aspect of non-existence of PMM2 performing an ideal inverse work-to-heat conversion cycle implies that it is not possible to convert a given amount of mechanical energy at high-pressure-low-volume into thermal energy with no production of mechanical energy at low-pressure-high-volume.
4. Thermal aspect of non-existence of PMM2 performing an ideal direct heat-to-work conversion cycle implies that it is not possible to convert a given amount of mechanical energy at low-pressure-high-volume into mechanical energy at high-pressure-low-volume with no contribution of thermal energy input.

### 5.2. Chemical-Mechanical PMM2

Chemical-mechanical aspect relating to mass-to-work or work-to-mass interactions conversion occurring in open systems:

5. Mechanical aspect of non-existence of PMM2 performing an ideal direct mass-to-work conversion cycle implies that it is not possible to convert a given amount of chemical energy at high potential into mechanical energy with no production of chemical energy at lower potential; in this case, the PMM2 canonical efficiency is $\eta^{\begin{array}{l} DIRECT\\ CHEMICAL \end{array}}=1-\frac{\mu_{R}}{\mu }$.
6. Chemical aspect of non-existence of PMM2 performing an ideal inverse work-to-mass conversion cycle implies that it is not possible to convert a given amount of chemical energy at low potential into chemical energy at high potential with no contribution of mechanical energy input.
7. Mechanical aspect of non-existence of PMM2 performing an ideal inverse work-to-mass conversion cycle implies that it is not possible to convert a given amount of mechanical energy at high-pressure-low-volume into chemical energy with no production of mechanical energy at low-pressure-high-volume.
8. Chemical aspect of non-existence of PMM2 performing an ideal direct mass-to-work conversion cycle implies that it is not possible to convert a given amount of mechanical energy at low-pressure-high-volume into mechanical energy at high-pressure-low-volume with no contribution of chemical energy input.

### 5.3. Physical Meaning of PMM2 Impossibility

One of consequences of the Second Law, or from a different perspective, the ultimate cause of irreversibility intrinsic to all processes, is the subdivision of systems configuration among levels of a hierarchical structure. In the special case of a molecule, once rigid constraints determining the whole mass to behave as a unique physical entity are removed, then the consequent distribution and dispersion of all components of internal energy is spread among increased available degrees of freedom of vibrating atoms or groups of atoms. The non-existence of Maxwell’s demon hence prevents to reverse any process attempting to bring the system back to its original configuration. As the existence of Maxwell’s demon is impossible, the reverse process from a lower to a higher hierarchical level is impossible as well since a PMM2 does not exist that is able to convert the entire amount of energy of a hierarchical level into energy of a higher level in the whole system configuration hierarchy.

The importance of a complete characterization of all types of PMM2 is ascribed to the fact that the impossibility of PMM2 is a corollary of Second Law and implies a cycle efficiency η<1 in any conversion processes. The ultimate cause of impossibility of PMM2 is the inter-particle collision characterized by the quantum uncertainty that determines microscopic irreversibility, as demonstrated by Lucia [33]. The not complete conversion of energy determines the available energy and, consequently, exergy used in the formulation of thermodynamic entropy and its components, as demonstrated in the following sections. By virtue of the intrinsic correlation between physical and informational content of systems and phenomena, the impossibility of PMM2 can be retrieved in any calculation process and device hence providing a proof of the Landauer’s principle [34,35,36,37].

## 6. Hierarchical Configuration and Levels of Multiscale Mesoscopic Systems

Thermodynamic systems can be regarded as a set of many-particles or few-particles as assumed in the framework of statistical physics and kinetic theory. Interactions among particles depend on relative velocity and relative position thus determining the kinetic energy and potential energy representing fundamental components of the internal energy characterizing any state of any system. The internal energy, at each available state, is subdivided in, and accommodated among, all translational and rotational degrees of freedom of the system which can be regarded as constituted by hierarchical levels with respect to the aggregation of masses of particles. The transitions among levels have been treated by Grmela et al. [38,39,40,41] in a rigorous and axiomatic mathematical formalism demonstrating the classical and quantum implication of entropy between two different levels accounting for non-equilibrium dissipative and non-dissipative dynamics. One of the major outcomes of Grmela analysis is that dissipative and non-dissipative dynamics are coupled in reduction and extension from one level to another one. On this basis, the attempt is here to investigate the possibility of specializing the definition of entropy by replicating its intrinsic structure for all coexisting levels of a hierarchical structure shaping the configuration of a multiscale mesoscopic system including quantum scale. In this perspective, physical and informational domain should remain a unique paradigm.

### 6.1. Maxwell’s Demon and Degrees of Freedom

As far as the hierarchical configuration is concerned, vibrational (translational and rotational) degrees of freedom are considered pertaining to a lower hierarchical level. Indeed, aggregates of particles, such as atoms bonded in a molecule or protons and neutrons bonded in the nucleus of an atom, behave differently from the same particles with no binding constraints. Then, removal of constraints determines an increase of degrees of freedom hence the configuration of the system has implications on equilibrium and non-equilibrium phenomena occurring within it. The main consequence is that, if particles are bounded to each other and constitute a rigid whole, then kinetic energy and potential energy of the rigid whole itself can be entirely transferred to the external system as work interaction. Instead, free independent particles of the same system with equal content of internal energy are not more able to transfer the entire amount of internal energy by means of work interaction to the external system. The ultimate reason of this limit is that a Maxwell’s demon does not exists that is capable to select positions and velocities of particles in such a way to move the system back to thermodynamic potentials characterizing non-equilibrium configurations.

### 6.2. Degrees of Freedom and Hierarchical Levels

The hierarchical configuration of thermodynamic systems, both many-particle of few-particle, is related to the geometric and kinematic framework of constraints and degrees of freedom determining properties and phenomena occurring along processes. In a unitary perspective, macroscopic and microscopic systems should behave consistently and laws not to be in contradiction. Thus, the relationship between the macroscopic (continuum) view of Classical Thermodynamics and its microscopic (particles) view as conceived in the framework of Classical and Quantum Statistical Thermodynamics, is here assumed as reported by Kline [42]. Differently from Classical Thermodynamics, the microscopic description of a system, in the fundamental assumptions and physical model proposed by Gibbs, is the ensemble constituted by number of replica $\bar{N}$ of a system containing $n_{i}$ particles. Gibbs ensembles are suitable to account for independent or dependent particles as occurring in solid state of matter, liquids or gases. The internal energy associated to each and every particle is distributed according to Gibbs canonical distribution of fractions $p_{i}=n_{i}/\bar{N}$ of molecules $n_{i}$ (out of the total amount of molecules N) in the state i $p_{i}=n_{i}/N=e^{-\beta \varepsilon_{i}}/\sum_{i}e^{-\beta \varepsilon_{i}}$ where $\beta =1/k_{B}T$ and $Q=\sum_{i}e^{-\beta \varepsilon_{i}}$ is the Gibbs canonical partition function derived from the statistical thermodynamic entropy formulation $S=k_{B}\ln W$ where $k_{B}$ is the Boltzmann constant and W is the number of different particles configurations. The partition function describes a configuration, in terms of positions and velocities of the phase space, resulting from the distribution of particle energies among the energy levels allowable for a given thermodynamic state of a microscopic system in stable equilibrium [5,6,43]. The relationship between macroscopic and microscopic representation model of a system is the rationale behind the conception of entropy defined in terms of degree of distribution of phenomena among the elements constituting a system [44]. The typical thermodynamic system considered in statistical physics is composed of molecules. However, each and every molecule is in turn composed of atoms constrained by electro-magnetic forces acting as chemical bonds. Atoms move in three translational and three rotational vibration modes while the molecule itself moves as a whole along its three translational and three rotational degrees of freedom. These two different modes (external dynamics and internal vibration) establish a hierarchical relationship within a molecule so that positions and velocities, at a higher Hierarchical Level (HL1) of the molecule as a whole, imply different thermodynamic properties with respect to a lower Hierarchical Level (HL2) where sub-molecules and atoms behave independently within their own degrees of freedom. Vibration modes of motion, in the perspective of translation and rotation at lower hierarchical level, should be considered as a consequence of constraints suitable: (i) to separate two hierarchical levels; and (ii) to allow relative velocities and displacements; hence, constraints fulfilling these requirements can only be interactions constituting dynamical correlations among particles. The increase of degrees of freedom and constraints has an impact on the content of information that the system accommodates and needs for the extraction of energy along any process. In this regard, a superpositions of multiscale systems modeling is adopted addressing to macro-level (macroscopic), meso-level (mesoscopic) micro-level (microscopic) and quantum-level (quantum-scopic) representing hierarchical levels implied in equilibrium and non-equilibrium thermodynamic processes. The importance and criticality of hierarchical models of systems in equilibrium or non-equilibrium is corroborated by current studies reported in the literature [38,39,40,41]. Referring to the energy levels available in a given state of the system defined in Statistical Physics, $\varepsilon_{i}=\varepsilon_{i}^{TRANSLAT}+\varepsilon_{i}^{ROTAT}+\varepsilon_{i}^{VIBRATION}$ should be regarded in the following breakdown:

(2) $$\varepsilon_{i}^{HL-HIGHER}=\varepsilon_{i}^{TRANSLAT}+\varepsilon_{i}^{ROTAT}$$

(3) $$\varepsilon_{i}^{HL-LOWER}=\varepsilon_{i}^{\begin{array}{l} VIBRATION\\ TRANSLAT \end{array}}+\varepsilon_{i}^{\begin{array}{l} VIBRATION\\ ROTAT \end{array}}$$

The above Equations (2) and (3) formally express the hierarchical paradigm deriving from the subdivision of a physical entity into interacting elements and the consequent degrees of freedom availability. The transition to subdivided particles occurs through stochastic change of Hamiltonian equation and is governed by same probabilistic mechanism as in intrinsic quantum collisions and particles motion dynamics.

The formal correspondence between Boltzmann-Gibbs Entropy and Shannon Information confirms the relationship between Statistical Physics and Information Theory. However, information is inherently associated to physical states of system and, on the other side, physical properties and phenomena inherently embed information. This bi-directional relationship constitutes the rationale to extend thermodynamic principles and properties, at any mesoscopic hierarchical level, to informational aspect of any system. Therefore, thermodynamic information (or information of thermodynamics) and informational thermodynamics (or thermodynamics of information) can be regarded as the two aspects of a thermodynamic-informational duality of the ultimate essence of any interaction (classical and quantum, non-statistical and statistical) occurring among any particle in any state. In this duality, an interaction, as a transfer of property, corresponds to communication as a transfer of information. The relationship is overarching any level of the hierarchy from classical to quantum scale. This fundamental fact concerns any portion of matter as a unique entity that is physical and informational and is accounted for by the existence of a generalized thermodynamic and informational entropy property.

## 7. Generalized Thermodynamic Entropy and Exergy Properties

A consequence of Second Law, and its corollary consisting of the non-existence of PMM2, is the definition of entropy property of a system A that, beside the classical formulation of Clausius $S_{1}-S_{0}=\int_{0}^{1}\frac{\delta Q}{T}$ in finite terms, has been expressed in the following form [1,2,3,4]: $S_{1}-S_{0}=\frac{1}{C_{R}}\left[\left(E_{1}-E_{0}\right)-\left(EX_{1}^{R}-EX_{0}^{R}\right)\right]$ where $C_{R}$ is a constant characterizing an external reference system R behaving as a reservoir, E is the internal energy determined by temperature, chemical potential and pressure, and $EX^{R}$ is the thermal exergy of the system. It has been proven that the thermodynamic entropy is an inherent property of any system, large or small, in any state, equilibrium or non-equilibrium [2,3]. Therefore, the reservoir behaves as an auxiliary system only [4]. Since the parameter characterizing the reservoir is the temperature, for this very reason the thermal exergy expresses the maximum net useful work available in the system-reservoir composite; then, this definition relates to the thermal entropy $S^{T}$.

### 7.1. Thermodynamic Entropy Components

As the thermodynamic entropy has been proved to be an inherent property of any system in any state, hence it has to relate to all forms of interactions with the external system R and therefore should result from the following components [11,12,13]:

(4) $$\mathrm{Thermal}\;\mathrm{Entropy}:{\left(S_{1}-S_{0}\right)}^{T}=\frac{1}{T_{R}}{\left[\left(E_{1}-E_{0}\right)-\left(EX_{1}^{R}-EX_{0}^{R}\right)\right]}^{T}$$

(5) $$\mathrm{Chemical}\;\mathrm{Entropy}:{\left(S_{1}-S_{0}\right)}^{C}=\frac{1}{\mu_{R}}{\left[\left(E_{1}-E_{0}\right)-\left(EX_{1}^{R}-EX_{0}^{R}\right)\right]}^{C}$$

(6) $$\mathrm{Mechanical}\;\mathrm{Entropy}:{\left(S_{1}-S_{0}\right)}^{M}=\frac{\bar{R}}{P_{R}V_{R}}{\left[\left(E_{1}-E_{0}\right)-\left(EX_{1}^{R}-EX_{0}^{R}\right)\right]}^{M}$$

Then entropy is derived from exergy and can be calculated based of the amount of work interaction along a so called “mechanical process” in which a mass undergoes displacements in the same direction of gravitational or electro-magnetic field force. All $S^{T},S^{C},S^{W}$ components of thermodynamic entropy above defined are extensive and additive properties as a consequence of their definition. Indeed, the additivity of entropy is a consequence of the additivity of energy and exergy appearing as terms of the entropy formulation [1]. In particular, exergy is directly derived from the generalized available energy that has been proved to be additive by virtue of the interaction of a system with the reservoir considered as the external reference system accounted for in its definition [1].

The concept of equivalence and interconvertibility demonstrated by Gaggioli [25,26,27], further corroborates the need of entropy contributions specially defined for thermal, chemical and mechanical forms of energy and interaction. To do so, the definition of generalized thermodynamic entropy consists of the sum of terms expressing thermal, chemical and mechanical contributions of entropy property being extensive and additive for any system in any state [29,30,31]:

(7) $$S^{G}=S^{T}+S^{C}+S^{M}$$

where $S^{T}$ is the thermal entropy, or kinematic entropy, $S^{C}$ is the chemical entropy, or geometric entropy, and $S^{W}$ is the mechanical entropy characterizing, respectively, heat, mass and work interactions with useful external system and non-useful external reservoir. The physical meaning of these contributions can be clarified in relation to the microscopic model of the system constituted by a set of few particles or many particles in the framework of Statistical Physics perspective. These contributions are characterized by inter-particle kinetic energy associated to particles relative velocity and inter-particle potential energy associated to particles relative position. Then, thermal entropy and chemical entropy represent, respectively, the degree of distribution of inter-particle kinetic energy and inter-particle potential energy among the degrees of freedom characterizing the system configuration at all hierarchical levels [44].

In addition, mechanical entropy accounts for the density of inter-particle kinetic energy caused by the collision frequency determined by the volume and the density of inter-particle potential energy caused by the repulsion intensity determined by the volume as well.

The configuration of any system and the hierarchy established by the set of constraints and degrees of freedom determines the hierarchical structure of entropy property as the consequence of the existence of hierarchical levels HL of the system partition. It is here assumed that entropy is a measure of the degree of sub-division of phenomena and properties among all accessible levels and degrees of freedom characterizing the hierarchical configuration of a system [44]. This dissipative sub-division process is intrinsically irreversible along non-equilibrium processes and through different levels according to Equations (2) and (3). This fact is reflected in the concept of entropy for non-equilibrium states [45,46] and non-equilibrium dynamics, as presented in different well-known theories such as the General Equation for Non-Equilibrium Reversible Irreversible Coupling (GENERIC) [39,40] and the Steepest-Entropy-Ascent (SEA) [47] are not at all discussed and their validity not criticized. On the contrary, these theories should be corroborated by their extension and application to configurations characterized by the stratification and superimposition of coexisting physical layers organized as hierarchical levels, in the sense here described, underpinning the complexity of multi-scale systems and considering the dualism ascribed to physical–informational character of matter. However, on an evolutionary time scale, the hierarchical architecture of complex systems is determined by the Maximum Entropy Production Principle [48] (overarching SEA and GENERIC) as the effect of Second Law acting on multi-level biotic systems evolution, as described by Annila [49].

**Figure 1:**
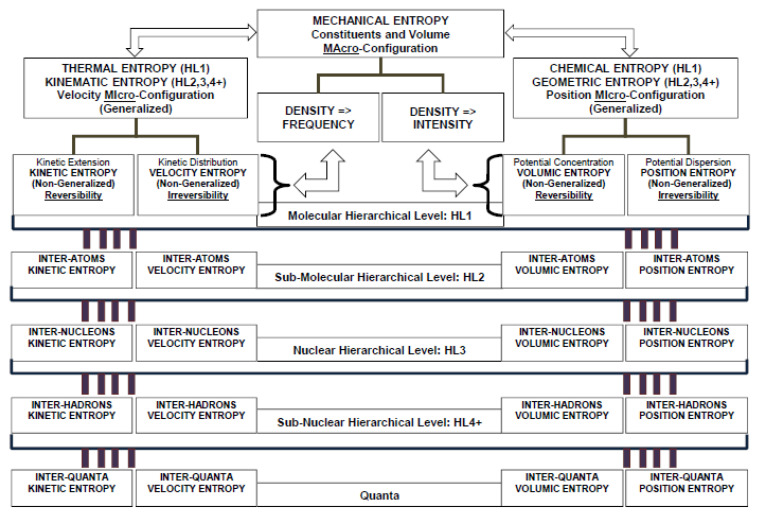
Hierarchical structure of generalized thermodynamic entropy property.

The non-existence of Maxwell’s demon represents the inherent physical limit preventing the upgrade of the entire amount of energy at a certain hierarchical level to a higher level. Hence, a hierarchical structure of entropy property definitions is founded on this intrinsic property and is represented in Figure 1.

The sum extended to all hierarchical levels leads to the generalized thermodynamic entropy expressed as:

(8) $$S^{G}=S_{HL1}^{G}+S_{HL2}^{G}+S_{HL3}^{G}+S_{HL4+}^{G}$$

The definition in terms of density and frequency spans from classical and statistical thermodynamics is adopted to describe the origin of energy and entropy contributions due to kinetic energy and potential energy of a microscopic system up to the quantum mechanics domain for the Schrodinger equation $\overset{\wedge }{H}\Psi =\left(\hbar^{2}/2m\right)\nabla^{2}\Psi +V\Psi =E\Psi$ is constituted by the Hamiltonian operator resulting from the sum of a kinetic operator and a potential operator of the wavefunction Ψ. This definition extends the hierarchical levels to the scale of atomic, nuclear and sub-nuclear systems where quantum mechanics provides the equations describing the motion of particles [7]. However, the ultimate origin of Second Law is rationalized in the conceptual paradigm of quantum thermodynamic that governs equilibrium and non-equilibrium processes at fundamental microscopic level. The irreversibility is inherent due to quantum states being “characterized by irreducible intrinsic probabilities” [8,9,10,11], and the physical entropy is an intrinsic and non-statistical property of matter. Steepest-entropy-ascent of microscopic dynamics is contextualized in quantum thermodynamics.

### 7.2. Exergy Contributions

Based on the entropy-exergy relationship, the generalized exergy property $EX^{G}$ can be defined in the canonical terms of maximum net useful interactions withdrawn from a system-reservoir composite [29,30,31]. The generalized exergy consists of the sum of thermal, chemical and mechanical contributions relating to each and every hierarchical level or, adopting the definitions here proposed, thermal exergy or kinematic exergy $EX^{T}$, chemical exergy or geometric exergy $EX^{C}$ and mechanical exergy $EX^{M}$:

(9) $$EX^{G}=EX^{T}+EX^{C}+EX^{M}$$

where $EX^{T}={\left(W_{10}^{AR\to }\right)}_{HEAT}^{MAX}$ is the maximum net useful work due to heat-to-work conversion direct cycle implying the minimum non-useful heat released to the reservoir; $EX^{C}={\left(W_{10}^{AR\to }\right)}_{MASS}^{MAX}$ is the maximum net useful work due to mass-to-work conversion direct ideal cycle implying the minimum non-useful mass released to the reservoir; $EX^{M}={\left(Q_{10}^{AR\to }\right)}_{WORK}^{MAX}$ is the maximum net useful heat due to work-to-heat conversion inverse ideal cycle implying the minimum non-useful work released to the reservoir; and $EX^{M}={\left(M_{10}^{AR\to }\right)}_{WORK}^{MAX}$ is the maximum net useful mass due to work-to-mass conversion inverse ideal cycle implying the minimum non-useful work released to the reservoir.

On the other hand, this expression applies to all hierarchical levels, then:

(10) $$EX^{G}=EX_{HL1}^{G}+EX_{HL2}^{G}+EX_{HL3}^{G}+EX_{HL4+}^{G}$$

Nuclear fission and fusion reactions Second Law analyses based on exergy method represent a possible application of these expressions. In the case of nuclear reactions, binding energies and kinetic energies of particles and nuclei fragments, associated to the mass defect, are accounted for to calculate all components of exergy and deriving the generalized thermodynamic entropy variation. In addition, fission and fusion represent processes of particles subdivision or assembling, respectively, between two different hierarchical levels. The inherent relationship between thermodynamic and informational aspect can be evaluated considering the modifications occurring to degrees of freedom and bonds involved in these reactions.

## 8. Hierarchical Structure of Thermodynamic Entropy and Exergy Properties

The geometric and kinematic configuration of a system at any state affects the hierarchical properties describing phenomena occurring among all particles and sub-particles. One of the most important consequences is that kinetic energy and potential energy at different hierarchical levels could not be characterized by the same “availability”. Once the kinetic energy or potential energy has been spread and subdivided into contributions pertaining to the degrees of freedom of a certain hierarchical level, then this amount of energy could not be transferred back to the whole molecule. Indeed, the non-existence of Maxwell’s demon prevents the internal linear-angular vibration kinetic energy to be entirely converted into translation-rotation kinetic energy of the particle behaving as a whole. While vibrating, this energy undergoes continuous transformation from kinetic energy into potential energy, and vice versa, along the vibration motion degrees of freedom and could not be entirely transferred back to the particle as a whole rigid body. This implies the irreversibility of dispersion of particles positions from a higher hierarchical level to a lower one. A similar conclusion holds for internal bond potential energy. Once again, a Maxwell’s demon does not exist that is capable of selecting and separating particles with higher bond potential energy from those particles with lower bond potential energy. Thus, internal bond potential energy cannot be entirely transferred back to the higher hierarchical level where the particles behave as a rigid whole. In turn, this implies the irreversibility of distribution of particles velocities from a higher hierarchical level to a lower one.

Considering hierarchical levels requires a clarification concerning the meaning of macroscopic and microscopic terms. Macroscopic is intended as the set of particles, even only one particle, constituting the system, contributing to the macroscopic parameters characterizing the whole system; neither the absolute or relative dimensions of system particles and container nor the dimensional scale difference or the number of particles, determine the meaning of macroscopic model of a system. Microscopic means that the parameter characterizing the system as a whole are generated and derived from the properties describing phenomena involving all particles constituting the system. Therefore, even one sphere interacting with its container, with same or different order of magnitude of dimensions, can be considered under a microscopic non-statistical or microscopic statistical perspective. Classical and quantum conceptual frameworks and methods apply to their own dimensions range of particles and containers considering the proper approximations and validity limitations stated by those theories.

If a system with a hierarchically structured configuration is considered, the internal energy can be expressed by means of all components contributing to the entire amount pertaining to a given state of the macroscopic system:

(11) $$U=U\left(P,T,\mu \right)=U^{M}\left(P\right)+U^{T}\left(T\right)+U^{C}\left(\mu \right)+\sum_{i=1}^{HLs}E_{HLi}^{KINETIC}+\sum_{i=1}^{HLs}E_{HLi}^{POTENTIAL}\;=-PV+TS+\mu n+\sum_{i=1}^{HLs}E_{HLi}^{KINETIC}+\sum_{i=1}^{HLs}E_{HLi}^{POTENTIAL}$$

P: determined by the kinetic energy and potential energy of particles per unit of volume;
T: kinetic energy per unit of particles; and
μ: potential energy per unit of mole.

The hierarchical levels breakdown structure is the following:

(a) Zeroth Hierarchical Level (HL0): The system is considered as a macroscopic rigid whole.
(b) First Molecular Hierarchical Level (HL1): Phase-Constituent, the macroscopic system is considered as a set of atoms and/or molecules; −PV (mechanical internal energy); translational–rotational kinetic energy; and translational–rotational potential energy.
(c) Second Sub-Molecular Hierarchical Level (HL2). Atoms and sub-molecules, as component elements of molecules, constitute a microscopic system: +TS+μn (vibration-translational/vibration-rotational kinetic energy and vibration-translational/vibration-rotational potential energy).
(d) Third Nuclear Hierarchical Level (HL3). Nucleons (protons and neutron) and electrons constitute atoms or group of atoms of group of molecules: $E_{HL3}^{KINETIC}+E_{HL3}^{POTENTIAL}$(vibration-translational/vibration-rotational kinetic energy and vibration-translational/vibration-rotational potential energy).
(e) Fourth Sub-Nuclear Hierarchical Level (HL4). Sub-nuclear hadrons and particles constitute a nucleus: $E_{HL4}^{KINETIC}+E_{HL4}^{POTENTIAL}$ (vibration-translational/vibration-rotational kinetic energy and vibration-translational/vibration-rotational potential energy).

Exergy is a non-conservative and additive state property. In the case of hierarchical structure of exergy, mentioned in the previous section, the generalized expression can be stated for any system characterized by hierarchical levels considering that exergy is an additive property:

(12) $$EX^{G}=EX_{HL1}^{G}+EX_{HL2}^{G}+EX_{HL3}^{G}+EX_{HL4}^{G}\;\mathrm{with}\;EX_{HL1}^{G}>EX_{HL2}^{G}>EX_{HL3}^{G}>EX_{HL4}^{G}$$

The above inequalities are the consequence of the relationship between the hierarchical structure of molecular configuration and entropy and exergy properties definition for equilibrium and non-equilibrium states. In fact, the generalized exergy represents the maximum net useful interaction (work, heat, mass), that can be extracted from all hierarchical levels of a system characterized by a hierarchical configuration with HLi levels.

As said, entropy is an inherent property of any system, large (many-particle) or small (few-particle) and is characterized by its own hierarchical configuration. Hence, nuclear energy is determined by nuclear entropy defined based on kinetic energy and potential energy distribution among all nucleons and particles constituting a single atom.

The point is that a machine does not exist which is capable to govern the process of progressive distribution of initial high density energy in a sub-system to be transferred as work interaction. In other terms, an elemental device, such as a cylinder-piston or electro-magnetic field, which is capable of collecting and transferring the inter-particle kinetic energy and potential energy to the external system via work interaction does not exist.

The transition from internal energy to external energy with respect to the external system implies an entropy increase due to irreversible conversion of external energy to internal energy that prevents the opposite process.

The definition of entropy property for a given hierarchical level $S_{HL}$ can be expressed considering the distribution of kinetic energy and potential energy among all degrees of freedom pertaining to that level:

(13) $$dS_{HL}^{KINEMATIC}=\frac{\delta Q^{HL}}{T^{HL}}=\frac{dE_{R}^{HL}}{T_{R}^{HL}}\;dS_{HL}^{GEOMETRIC}=\frac{\delta M^{HL}}{\mu^{HL}}=\frac{dE_{R}^{HL}}{\mu_{R}^{HL}}$$

and, in the generalized form extended to all types of interactions:

(14) $$dS^{G}=\frac{\delta I^{G}}{P^{G}}=\frac{dE_{R}^{G}}{P_{R}^{G}}=\frac{d\left(E^{G}-EX^{G}\right)}{P_{R}^{G}}$$

where $P^{G}$ and $P_{R}^{G}$ represent the generalized potential of system and reservoir, respectively.

### 8.1. Macroscopic Level

At macroscopic level, the expression of entropy property, derived from energy and exergy properties, can be applied to a microscopic system in which few particles interact, as in the case of fission or fusion nuclear reactions:

(15) $${\left(S_{1}^{MECHANICAL}-S_{0}^{MECHANICAL}\right)}^{HL}=\frac{\bar{R}}{P_{R}^{HL}V_{R}^{HL}}{\left[{\left(E_{1}-E_{0}\right)}^{M}-{\left(EX_{1}^{R}-EX_{0}^{R}\right)}^{M}\right]}^{HL}$$

(16) $${\left(S_{1}^{KINEMATIC}-S_{0}^{KINEMATIC}\right)}^{HL}=\frac{1}{T_{R}^{HL}}{\left[{\left(E_{1}-E_{0}\right)}^{K}-{\left(EX_{1}^{R}-EX_{0}^{R}\right)}^{K}\right]}^{HL}$$

(17) $${\left(S_{1}^{GEOMETRIC}-S_{0}^{GEOMETRIC}\right)}^{HL}=\frac{1}{\mu_{R}^{HL}}{\left[{\left(E_{1}-E_{0}\right)}^{P}-{\left(EX_{1}^{R}-EX_{0}^{R}\right)}^{P}\right]}^{HL}$$

in which $T_{R}^{HL}$ represents the temperature and $\mu_{R}^{HL}$ the potential of the reservoir considered as an auxiliary reference system.

It is noteworthy that the thermodynamic state of the reservoir does not affect the entropy content at any hierarchical level since the reservoir is an auxiliary reference system only, therefore a unique reservoir can be considered as the reference system for all hierarchical levels.

As the above definition of entropy property is to be considered valid for many-particle or few-particle, this expression has to be valid for few particles involved in the particular case of nuclear reactions. To do so, the calculation of energy and exergy would allow calculating the three contributions of entropy property.

### 8.2. Microscopic Level

At microscopic level, the classical statistical mechanics and thermodynamics describe the system in terms of substructures by means of the method established by Gibbs for dependent and distinguishable particles whose expression of statistical canonical thermodynamic entropy property is the following expression in which positions and velocities in the phase space are identified:

(18) $$S=k_{B}\ln W=k_{B}\ln W^{p}+k_{B}\ln W^{q}=k_{B}N\sum_{i}\frac{n_{i}}{N}\ln\frac{n_{i}}{N}=k_{B}N\sum_{i}w_{i}\ln w_{i}$$

where $k_{B}$ is the Boltzmann constant and $k_{B}=\frac{\bar{R}}{N_{A}}$ where $N_{A}$ is the Avogadro number. N is the number of particles constituting the system. W is the weight of the most probable microscopic configuration of the system determining the macroscopic state. The weight is the number of different microscopic configurations corresponding to the number of possible distributions of particles among the particle kinetic and potential energy levels available at a given state. $w_{i}$ represents the fraction of particles in the state i out of all states corresponding to all different kinematic configurations and geometric configurations.

Being the phase space composed by coordinates related to both velocities $p_{n}$ and positions $q_{n}$ of each and every particle n, then two different expressions can be stated, for both contributions, as follows:

(19) $$S_{HL}^{KINEMATIC}=k_{B}\log W^{p}=k_{B}\sum_{i}N_{i}p_{i}^{p}\ln p_{i}^{p}$$

representing the Kinematic Entropy associated to the fraction $p_{i}^{p}$ of particles in the kinematic state i related to the velocity phase space; and

(20) $$S_{HL}^{GEOMETRIC}=k_{B}\log W^{q}=k_{B}\sum_{j}N_{j}p_{j}^{q}\ln p_{j}^{q}$$

representing the Geometric Entropy associated to the fraction $p_{j}^{q}$ of particles in the geometric state j related to the position phase space. This term is here adopted to include the “configurational entropy”, related to particles position in the phase space, and the “conformational entropy” addressing to all possible arrangements of complex molecules as occurring in macromolecules involved in biological systems; being entropy an extensive and additive state property, then the sum of different contributions can be expressed as:

(21) $$S_{HL}=S_{HL}^{KINEMATIC}+S_{HL}^{GEOMETRIC}=k_{B}N\sum_{i}\left(p_{i}^{p}\ln p_{i}^{p}+p_{j}^{q}\ln p_{j}^{q}\right)$$

The transition from a certain level to a lower level implies a (quantum) increase of the entropy pertaining to the starting level and therefore can be defined as the entropy of entropy. This recursive term originates from the cascade dissipation of energy along the progressive subdivision descending levels through the hierarchical configuration of systems. Mathematically, the formal expression of generalized entropy, considering that entropy is a non-conservative and additive state property, is given by:

(22) $$S^{G}=S_{HL1}^{G}+S_{HL2}^{G}+S_{HL3}^{G}+S_{HL4+}^{G}\;\mathrm{with}\;S_{HL1}^{G}<S_{HL2}^{G}<S_{HL3}^{G}<S_{HL4+}^{G}$$

## 9. Non-Equipartition Theorem of Entropy

The inter-dependency between Statistical Mechanics and Kinetic Theory suggests the correlation between the Boltzmann molecular distribution function and the equipartition theorem of energy for a system in a stable equilibrium state.

A many-particle or few-particle system constituted, in the most general case, by three-dimensional complex molecules can be described adopting the phase space in which positions and velocities of particles are analytically represented. The distribution of the total amount of energy of the system occurs, for an individual particle, among the following degrees of freedom: 3 rigid-translational + 3 rigid-rotational = 6. These degrees of freedom accommodate the inter-molecule potential energy depending on relative positions, and inter-molecule kinetic energy depending on relative velocity. In addition, each and every molecule is allowed 3 vibro-translational + 3 vibro-rotational = 6 degrees of freedom, constituting a lower hierarchical level, once again characterized by relative (inter-atomic) positions and velocities. The equipartition theorem of energy [5,6,43] establishes that the total amount of internal energy is spread among all available degrees of freedom and all levels of the system hierarchical configuration; moreover, one degree of freedom accommodates a portion of internal energy equal to $k_{B}T/2$ where $k_{B}$ is the Boltzmann constant and T is the absolute temperature. Consequently, the equipartition of energy implies that each and every degree of freedom accommodates an equal portion of overall internal energy content of a system accounting for all kind of energy that the system experiences. However, those degrees of freedom, pertaining to lower hierarchical levels associated to the internal configuration of particles, determine an irreversible degradation of properties characterizing the system.

Non-existence of Maxwell’s demon implies that equipartition of energy among degrees of freedom and therefore between two given hierarchical levels does not allow to bring back the whole amount of energy to upper levels. Indeed, no being or device is able to select molecules velocity or position to invert the system configuration. The amount of energy available to be transferred to the upper hierarchical level is the portion converted along a conversion cycle operating between two constant inter-particle temperatures, namely $T^{HLi}$ and $T^{HLi+1}$ or between two constant inter-particle potential namely $\mu^{HLi}$ and $\mu^{HLi+1}$. If multiple levels are accounted for, the final available energy as maximum net useful work at the upper level under consideration, resulting from conversion of heat interaction or mass interaction, is given by:

(23) $$W_{HL}=Q\prod_{i=0}^{i=HL}\eta^{HLi}\;\mathrm{and}\;W_{HL}=M\prod_{i=0}^{i=HL}\eta^{HLi}$$

A consequence of the hierarchical structure of systems is that kinetic energy and potential energy are equally distributed among all hierarchical levels and their degrees of freedom. Instead, entropy property is not equally distributed along whatever chemical and nuclear processes. Indeed, as a Maxwell demon does not exist, energy associated with a degree of freedom at a lower hierarchical level could not be entirely transferred to a higher hierarchical level. Therefore, if the overall content of energy is equally distributed among a higher number of hierarchical levels and lower degrees of freedom corresponding to each level, then the amount of energy available to be transferred by work interaction to a weight process is lower. This irreversibility, related to the Second Law, is the essence of the non-equipartition theorem of entropy which would be complementary to the equipartition theorem of energy derived from the First Law. The non-equipartition implies the maximum entropy principle at each hierarchical level and a proposal has been already elaborated for superstatistical systems [50].

As far as processes are concerned, even equilibrium states can determine irreversible process in case a lower hierarchical level is implied in the interactions among different portions of the system.

The non-equipartition theorem of entropy is determined by systems configuration subdivision relating to degrees of freedom and constraints among constituting particles and reflects the fact that distributed and dispersed systems maximize entropy along irreversible processes. The spontaneous tendency of these processes is to extend to all levels of coexisting hierarchical structures, nested in any system, reversible and irreversible phenomena along steady conservative or non-conservative processes [48]. Instead, in the opposite direction, the tendency to maximize energy dissipation in non-equilibrium processes induces the system evolution to generate hierarchically structured configurations [49]. However, a morphogenetic counter-tendency appears along transient constructive evolution as in the constructional theory [51,52,53,54] and the entropy generation minimization method [55] representing the driving project of systems architecture shaping, parts assembling and matter aggregation. The following logical relations between these paradigms are established for equilibrium or non-equilibrium phenomena:

- Equipartition theorem of energy: Reversible and irreversible conversion processes and maximum entropy production principle ⇒ multi-scale configuration of systems emerging from energy dissipation along hierarchical levels.
- Non-equipartition theorem of entropy: Reversible and irreversible conversion processes and entropy generation minimization paradigm ⇒ constructive evolution of systems through self-organizing capability and shaping of optimized hierarchical configurations.

Hence, the constructive evolution would describe the complementary trend of thermodynamic and informational phenomena occurring in a system, in the opposite sense with respect to the Non-Equipartition Theorem of Entropy centered on its physical and informational significance implied in hierarchical configuration of systems driven by dissipation processes.

Future developments may envisage applications of the methodologies here discussed to biotechnologies or nanotechnologies and nanosystems [56,57,58] in which the self-assembling and self-organizing capabilities are used as tools to govern matter manipulation.

## 10. Conclusions

The present research illustrates the concept that the dualism and the relation arising from thermodynamic and informational aspect of entropy property play a fundamental role in matter and phenomena description of multi-scale systems characterized by hierarchical configurations. This conceptual schema, underpinned by the physical nature of information and the informational content of physical states, is recognized as inherent to any system and provides an overarching and unitary perspective over the domain from classical through statistical to quantum physics. The non-existence of Maxwell’s demon, implied with the Second Law, represents the fundamental rationale behind the hierarchical levels definition and analysis. The Second Law statement has been specialized for ideal conversion cycles governed by chemical potentials to further extend the common approach based on temperatures. Consequently, an extension of a corollary of the Second Law, consisting of the impossibility of PMM2, to all thermal–mechanical and chemical-mechanical conversion processes, is a further result here outlined. The definition of generalized thermodynamic entropy and exergy properties have been proposed with the intent of extending to all hierarchical levels constituting the system configuration thus implying the calculation of entropy and exergy balance and efficiency in Second Law analyses. Finally, the irreversibility associated to the hierarchical configuration of a system, related to the Second Law, is the essence of the non-equipartition theorem of entropy which would be complementary to the equipartition theorem of energy derived from the First Law. A counter-tendency is revealed by the capability of systems in displaying constructive evolutive shaping of structures in the direction of entropy generation minimization opposite to the maximum entropy production determining hierarchical configurations of multi-level structures.
